# A Facile and Specific Assay for Quantifying MicroRNA by an Optimized RT-qPCR Approach

**DOI:** 10.1371/journal.pone.0046890

**Published:** 2012-10-05

**Authors:** Qian Mei, Xiang Li, Yuanguang Meng, Zhiqiang Wu, Mingzhou Guo, Yali Zhao, Xiaobing Fu, Weidong Han

**Affiliations:** 1 Department of Molecular Biology, Institute of Basic Medicine, School of Life Sciences, Chinese PLA General Hospital, Beijing, People’s Republic of China; 2 Department of Gynecologic Oncology, Institute of Basic Medicine, School of Life Sciences, Chinese PLA General Hospital, Beijing, People’s Republic of China; 3 Department of Cancer Epigenetics, Institute of Basic Medicine, School of Life Sciences, Chinese PLA General Hospital, Beijing, People’s Republic of China; Duke University, United States of America

## Abstract

**Background:**

The spatiotemporal expression patterns of microRNAs (miRNAs) are important to the verification of their predicted function. RT-qPCR is the accepted technique for the quantification of miRNA expression; however, stem-loop RT-PCR and poly(T)-adapter assay, the two most frequently used methods, are not very convenient in practice and have poor specificity, respectively.

**Results:**

We have developed an optimal approach that integrates these two methods and allows specific and rapid detection of tiny amounts of sample RNA and reduces costs relative to other techniques. miRNAs of the same sample are polyuridylated and reverse transcribed into cDNAs using a universal poly(A)-stem-loop RT primer and then used as templates for SYBR® Green real-time PCR. The technique has a dynamic range of eight orders of magnitude with a sensitivity of up to 0.2 fM miRNA or as little as 10 pg of total RNA. Virtually no cross-reaction is observed among the closely-related miRNA family members and with miRNAs that have only a single nucleotide difference in this highly specific assay. The spatial constraint of the stem-loop structure of the modified RT primer allowed detection of miRNAs directly from cell lysates without laborious total RNA isolation, and the poly(U) tail made it possible to use multiplex RT reactions of mRNA and miRNAs in the same run.

**Conclusions:**

The cost-effective RT-qPCR of miRNAs with poly(A)-stem-loop RT primer is simple to perform and highly specific, which is especially important for samples that are precious and/or difficult to obtain.

## Background

Recently, microRNAs (miRNAs) have been discovered in animals and plants. These non-coding RNAs, with a length of 19–25 nucleotides [1], [2], regulate gene expression at the post-transcription level via specific complementary sites within the 3′-untranslated region (UTR) of the target mRNAs, causing translational repression or degradation [3]. miRNAs have been reported to be major modulators in several cellular and pathological processes [4], including angiogenesis [5], apoptosis [6], cell cycle [7], proliferation [8], telomerase activity [9], and so on. They are believed to play a key role in human diseases, especially in tumorigenesis, invasion and metastasis [10].

Recently, research on miRNAs has increased sharply due to the growing awareness of their importance. An altered miRNA expression profile has been related to the developmental lineage and differential tumor states [10]. Although miRNAs represent a relatively abundant class of transcripts, the level of expression varies greatly among species and types of tissue [11]. Several methods have been employed to detect the expression of miRNAs in a variety of biological samples, but less abundant miRNAs often escape detection by technologies such as cloning, northern blot analysis [12] and microarray [13]. A novel real-time quantification method for the reliable and sensitive detection of mature miRNAs was proposed by Chen *et al.* in 2005 [14]. The stem-loop RT-qPCR assay (TaqMan® small RNA assays) has a high degree of sensitivity and a broad dynamic range in detecting the expression of miRNAs; however, it requires a primer specific for each species of miRNA molecule, so each miRNA molecule of every sample requires a RT reaction. Thus, it is difficult to obtain sufficient amount of samples to meet the huge requirements of this rather costly assay [15]. Shi R. and Chiang V. L. have developed another RT-qPCR protocol to detect the mature miRNA molecules using poly(T)-adapter primers [16]. This method (miScript™ PCR system) uses a universal primer for the RT reaction and thus needs only tiny samples, but the linear structure does not prevent binding to double-stranded genomic DNA. Furthermore, the Exiqon miRCURY LNA Universal RT microRNA PCR was developed to increase the Tm and the specificity by spiking the PCR primers with Locked Nucleic Acid (LNA). However, it has been reported that sequences containing LNA are poor templates for most DNA polymerases and decrease the amplification efficiency [17], [18].

We integrate and optimize the above current approaches, and present a cost-effective, more convenient, highly sensitive and accurate RT-qPCR method for the quantification of mature miRNA molecules.

## Results

### General Assay Design

**Figure 1:**
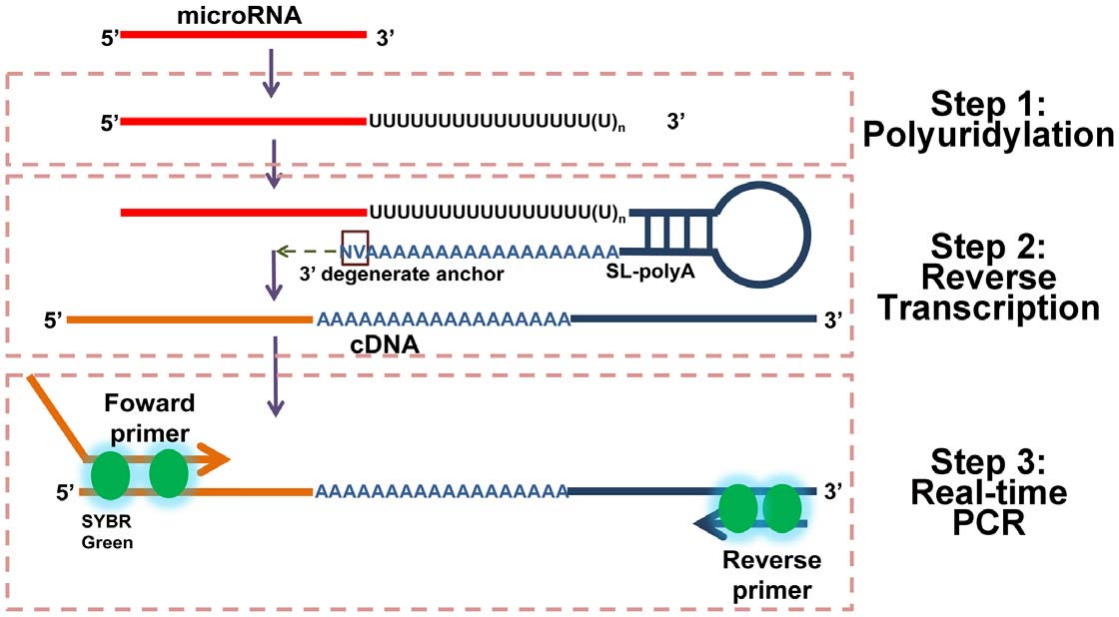
Schematic description of assay design. Real-time quantification of miRNA includes three steps: polyuridylation, RT reaction and real-time PCR. miRNAs are given a poly(U) tail by poly(U) polymerase. The cDNA molecules are reverse transcribed using poly(A) stem-loop RT primers (SL-poly(A)). The primer has 3′ degenerated anchor sequence and 18 nt of As. Such design guarantees an efficient priming of more than 18 nt U tailed miRNA. The miRNA-specific forward primer and the universal reverse primer are complementary to the stem-loop adapter used for the analysis.

A novel RT-qPCR scheme is proposed for the quantification of miRNA (Figure 1). The scheme consists of three steps: polyuridylation, RT reaction and real-time PCR. Initially, total RNA is polyuridylated with UTP by poly(U) polymerase. Polyuridylation is a random process, and usually results in over hundreds of U residue added to 3′ of RNA. Subsequently, cDNA molecules are reverse transcribed using the universal poly(A) stem-loop RT primers (SL-poly(A)). The SL-poly(A) primer comprises two main sequence portions: the 3′-poly(A) end for binding to the poly(U) tail of miRNA and the 5′-stem-loop end to provide a spatial constraint. The 3′-poly(A) end segment is similar with the miScript™ PCR system that is typical anchor primer with 3′ degenerated anchor sequence and 18 nt of As. This structure would provide the control of the stretch of U(s) to be included between the 3′end of the miRNA and the 5′end of the stem loop primer, and undertake an efficient priming of more than 18 nt U tailed miRNA. The stem-loop portion provides a binding site for the universal reverse primer of real-time PCR. A miRNA-specific forward primer and a universal reverse primer are used for the subsequent qPCR step. The 3′-end of the forward primer is miRNA-specific and is hybridized to the cDNA molecule of the miRNA of interest. A tail is present at the 5′-end of the forward primer increasing the melting temperature (T_m_) depending on the sequence composition of the miRNA.

The final quantification of miRNA is performed by real-time PCR analysis using the SYBR® Green fluorescence utilizing the 2^−ΔΔ*C*q^ method.

### Assay Sensitivity and Dynamic Range

**Figure 2:**
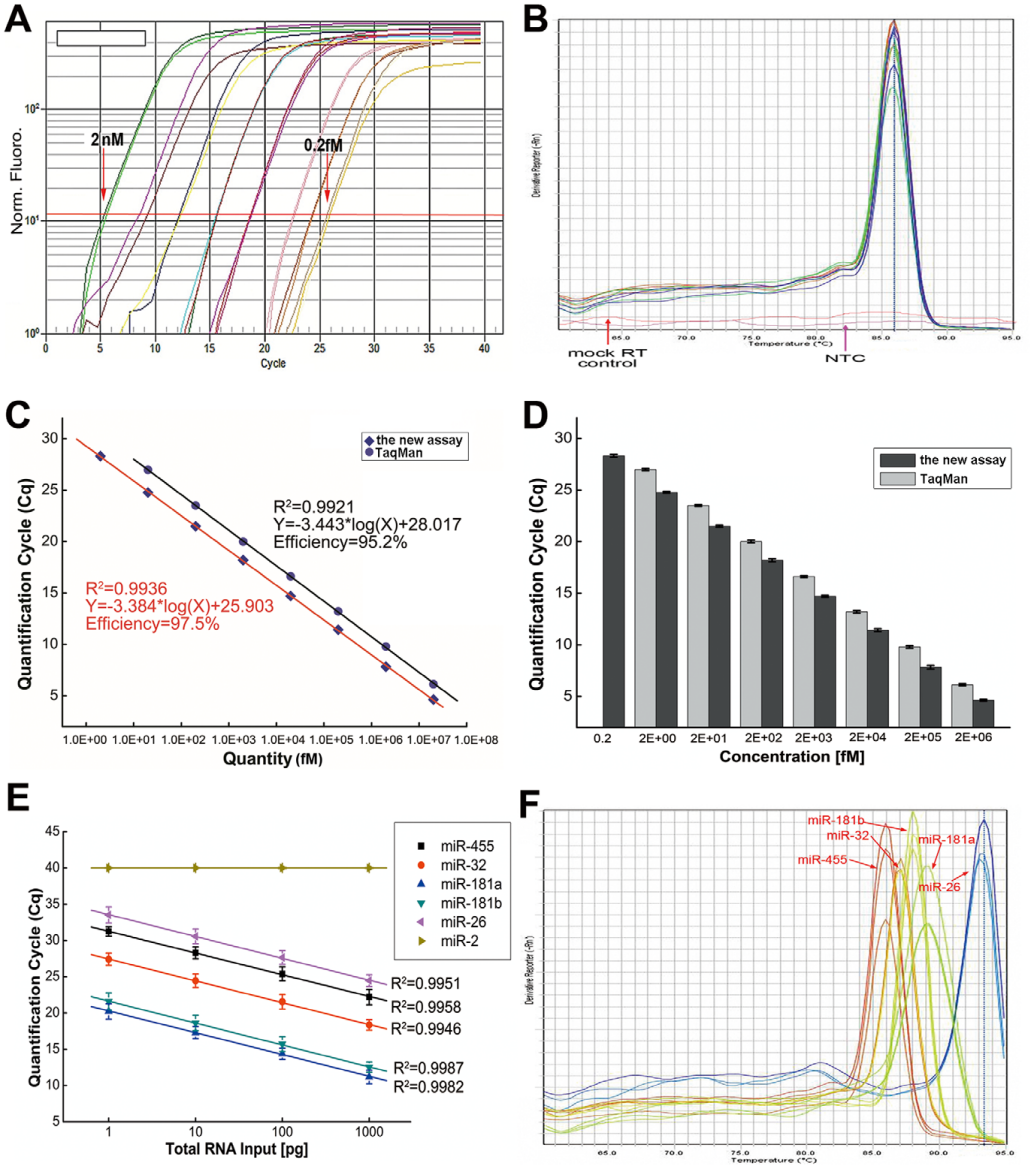
Dynamic range and sensitivity of the miR-32 assay. (A) Amplification plot of synthetic hsa-miR-32 miRNA. Target input ranged over eight orders of magnitude (0.2 fM–2 nM). (B) Melting curve of the miR-32 assay. Neither the mock RT control, nor the no-template control (NTC) showed a background signal over 40 cycles. (C) Standard curve of miR-32 of the new assay and TaqMan method. Curve of the new assay was a straight line (*R^2^* = 0.9936) with a slope of −3.384 (PCR efficiency = 97.5%) over eight orders of magnitude of the template. Curve of TaqMan method was also a straight line (*R^2^* = 0.9921) with slope of −3.443 (PCR efficiency = 95.2%) over seven orders of magnitude of the template. (D) The TaqMan method showed a sensitivity limit of 2 fM synthetic miR-32, while the sensitivity limit of the new assay turned out to be 0.2 fM synthetic miR-32. Each column represents the mean (± SD) of three measurements. (E) The *C*_q_ values of the miRNA assay correlated with the total RNA input. The total RNA of the SiHa cell input ranged from 1 to 1000 pg per RT reaction. Each value represents the mean (± SD) of three measurements. *Caenorhabditis elegans* miRNA (miR-2) was included as a negative control. (F) Dissociation curve analysis of the same experiment in panel E.

The dynamic range and sensitivity of the scheme were first evaluated using a synthetic miR-32 target. Synthetic microRNA was quantified based on the *A*_260_ value. A number of quantities of synthetic miR-32 (0.2 fM-2 nM in the RT reaction) were applied to analyze the dynamic range of the approach. The assay was performed using 50 ng yeast tRNA as the RNA carrier to increase the total RNA amount and the RNA complexity. The miR-32 assay exhibited fairly good linearity between the log of the target input and *C*_q_ values over eight orders of magnitude, detecting as little as 0.2 fM synthetic miR-32 (Figure 2A). The dissociation curve showing one peak from the qPCR amplification demonstrated the specificity of the reaction (Figure 2B). The assay exhibited high levels of specificity and sensitivity for miR-32; no background signal was seen over 40 cycles in the NTC (no-template control) and mock RT controls (RT reaction carried out only with carrier RNA) (Figure 2B).

As the most widely accepted approach, Chen’s method (TaqMan® small RNA assays) was applied to validate the sensitivity and quantitative of the proposed assay using 50 ng of yeast tRNA spiked with synthetic miR-32 to give final concentrations between 0.2 fM and 2 nM in the RT reaction. Both methods were performed within the same qPCR run. Amplification efficiency of the new approach (0.975) was comparable to the TaqMan assay (0.952), and correlation coefficient (R^2^) of both method were greater than 0.99 (Figure 2C). Four more miRNA assays were performed for futher comparison, and gave comparable results (Figure S1). Regardless of the two different assay conditions, the new approach provided a higher sensitivity (0.2–0.5 fM) compared with Chen’s method (2–5 fM), characterised by slightly lower Ct values and a higher dynamic range (Figure 2D and Figure S1C).

Further validation of the exhibited miRNA quantification protocol was obtained by using total RNA from SiHa cells. In this experiment, 1–1000 pg of total RNA was tailed with poly(U) and cDNA was transcribed from the poly(U)-tailed total RNA. The product was used as a template for SYBR® Green real-time PCR. The *C*_q_ values were correlated with the total RNA input (*R* ^2^>0.996) (Figure 2E). The cel-miR-2 negative control did not produce a detectable signal. The dissociation curve exhibited only one peak of each tested miRNA (Figure 2F). The analysis of a variety of samples revealed that the template quantity corresponding to 1–1000 pg of total RNA yielded optimal amplification efficiency and specificity (data not shown).

Moreover, there had been reports that indicated that the Exiqon miRCURY assay, which also uses poly(A) tailing of the miRNAs, increased specificity utilizing LNA, but lead to a decrease in amplification efficiency [17], [18]. To verify the amplification efficiency of the new assay, the amplification efficiencies of 10 miRNAs from SiHa cells were measured both with Exiqon miRCURY and the proposed assay. Amplification efficiency of the new assay (ranged from 90% to 105%) was higher as compared with the Exiqon miRCURY method (85–95%) (student’s *t*-test, P<0.05) (Table 1).

**Table 1:** Comparison of amplification efficiency of proposed assay with miRCURY method on SiHa cell total RNA.

microRNA	proposed assay (%)	miRCURY (%)	difference (%)
miR-455	96	93	2.8
miR-32	99	91	7.9
miR-181a	97	89	8.1
miR-181b	98	91	6.8
miR-126	92	87	9.2
let-7a	98	95	3.1
let-7b	90	86	4.2
let-7c	105	88	16.7
let-7d	101	92	9.2
let-7f	94	85	8.9
**average (SD)**	97 (4.3)	90 (3.2)	7.7 (4.0)

### Effect of Double-stranded DNA on the Assay

**Figure 3:**
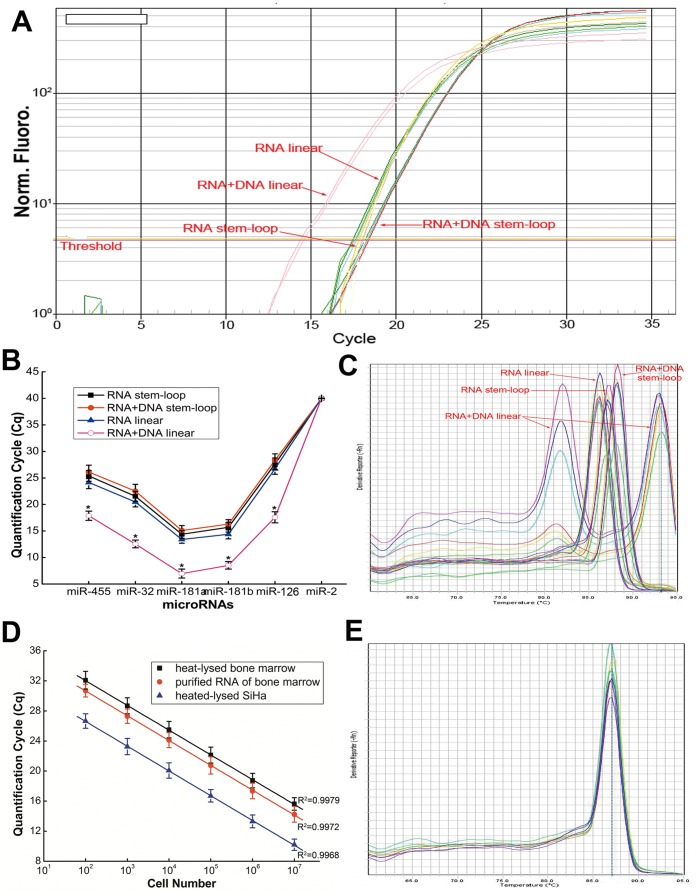
Effect of double-stranded genomic DNA on the miRNA assay. (A) Amplification plot of hsa-miR-32 miRNA from SiHa cell total RNA with and without double-stranded genomic DNA using stem-loop or linear adapter RT primer. (B) Comparison with and without genomic DNA in the two RT reaction systems for real-time quantification of 6 miRNAs. (C) Dissociation curve analysis of the same experiment of panel A. (D) Comparison of heat-treated SiHa cells, heat-treated bone marrow and purified total RNA of bone marrow for real-time quantification of hsa-miR-32 miRNA. (E) Dissociation curve analysis of the same experiment of panel D. (B & D) The level of miRNA expression is measured in the quantification cycle (*C*_q_). Each value represents the mean (± SD) of three measurements.

Chen *et al.* had observed the constraint of binding double-stranded genomic DNA owing to the stem-loop structure. To verify this effect in our assay, 5 ng of SiHa cell genomic DNA was added to the total RNA as a template. The *C*_q_ value showed no significant difference with or without the presence of genomic DNA (student’s *t*-test, P>0.05) (Figure 3A & B), and produced one peak during the melting curve analysis, respectively (Figure 3C). In contrast, the linear adapter (miScript) gave a different result; double-stranded DNA had a significant effect on the *C*_q_ values (Figure 3A & B) forming two peaks in the melting curve analysis (Figure 3C).

For ulterior validation and application, 10^2^–10^7^ cells of the bone marrow samples and SiHa cells wereheat-lysed as described in the Methods, and added directly as the substrate for polyuridination. We found a significant correlation between the *C*_q_ values and the added cell number (*R^2^*>0.993) (Figure 3D). Melting curve analysis produced only one peak (Figure 3E). Figure 3D presented good concordance of the *C*_q_ values between the purified total RNA and heat-treated derived from an equal number of bone marrow cells. The consistency of the two sample preparation methods shed light on the applicability of this approach in handling precious samples.

### Assay Specificity and Cross-reaction

**Figure 4:**
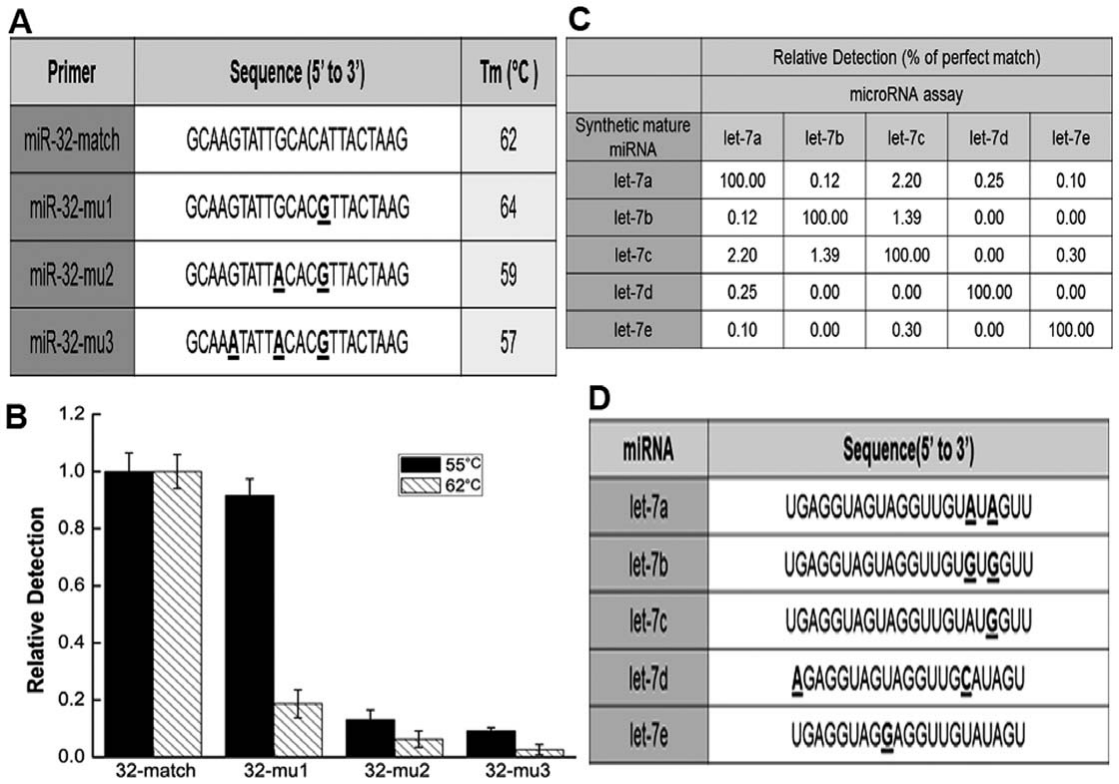
Specificity and cross-reaction analysis of the miRNA assay. (A&B) Relative level of PCR products using a mismatched primer compared to the perfectly matched primer in the normal program (annealing temperature 55°C) and in the high-stringency program (annealing temperature 62°C) for amplifying the target hsa-miR-32. Each column represents the mean (± SD) of three measurements. (C&D) Cross-reaction of the human let-7 family assays (annealing temperature 56°C). The percentage of cross-reaction values was calculated based on the *C*_q_ difference between assay-specific and nonspecific miRNA targets. The PCR annealing temperature was raised to 60°C to distinguish let-7a and let-7c.

The sequences of the miRNA paralogs are identical except for 1–3 mismatched bases. To evaluate the specificity of our real-time PCR approach, we tested the amplification with primers having perfect complementarity or with 1–3 nucleotides mismatched to the miR-32 sequence (Figure 4A). The primers with two or three mismatched bases did not result in products of significant levels (Figure 4B, mu2 and mu3), but primers with a single mismatched nucleotide led to almost the same extensive product amplification as perfectly matched primers (Figure 4B, mu1 and match). To modify the annealing efficient, we raised the PCR annealing temperature to 62°C, which is higher than the Tm value of single nucleotide mismatched primers. As a result, the modified PCR amplification level was reduced significantly (Figure 4B). These results attested that the specificity of the new miRNA quantification assay is favorable, but discrimination of miRNAs differing by a single base requires more stringent conditions.

The specificity of the present assay was ulteriorly assessed with inherent miRNA family. The let-7 family is a representative miRNA family with members that have similar sequences. Cross-reaction of five closely sequence-related members of the let-7 family (let-7a, let-7b, let-7c, let-7d and let-7e) differing in at least one nucleotides were employed for further analysis of the proposed approach (Figure 4C). Relative detection efficiency was calculated from differences of *C*_q_ between perfectly matched and mismatched targets, assuming 100% efficiency for the perfect match. The new assay displayed a high capability to discriminate between miRNA molecules, which differ by two or three bases (ranging from 0% to 0.3%). Marginal cross reaction was observed mainly at miRNAs that differed by a single nucleotide with minute values ranging from 0.1% to 2.2%. Only the targeted miRNA was detected if more than three mismatched bases between any two miRNAs were present (Figure 4D). Most cross-reactions resulted from let-7a assay versus let-7c as a target. So the forward primers of the PCR needed to meet special requirements to distinguish between let-7a and let-7c. These two forward primers have only one mismatched base at the 3′-end. Therefore, to improve the specificity of the assay, the forward primers of the PCR were designed to contain mismatched base, and the PCR annealing temperature was raised to 60°C.

### Advantage of Poly(U) Tail

**Figure 5:**
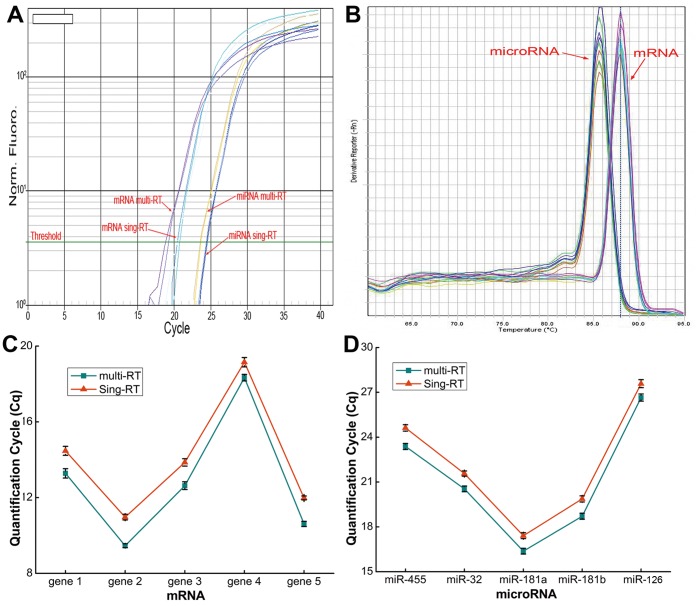
Advantage of U-tailing of miRNA. Both mRNA and miRNAs showed no significant difference of *C*_q_ value and dissociation curve based on multiplex or single RT reactions (P>0.05). mRNA and miRNAs did not affect each other in the same reaction system. **multi-RT**: both mRNA and miRNAs were reverse transcribed in a single RT reaction; **sing-RT**: mRNA and miRNAs were reverse transcribed in different RT reactions, respectively. Each value represents the mean (± SD) of three measurements.

We used a poly(U) tail instead of the usual poly(A) tail, and it provided more convenience and specificity. miRNAs were tailed by poly(U) and lacked the poly(A) tail, and therefore would not anneal to the ordinary oligo(T) RT primer. Conversely, the poly(A) tail of the mRNA was still present, and could bind to the oligo(T) RT primer. To prove the advantage of the U-tailing, we performed a multiplex RT reaction of mRNA and miRNAs within the same run. As shown in Figure 5, mRNA existence did not alter *C*_q_ values of microRNAs (student’s *t*-test, P>0.05), indicating that mRNA and miRNAs did not affect each other when combined in the same RT reaction. The dissociation curve showed a similar situation. Many commercially available strategies of mRNA RT reactions exploit the characteristics of the mRNA-poly(A) tail. The employment of poly(U) tail of miRNAs maked it convenient to use these strategies in the quantification of mRNA of target genes and miRNAs in the same system.

### miRNA Expression Profile of Four miRNAs in Mouse Tissues

**Figure 6:**
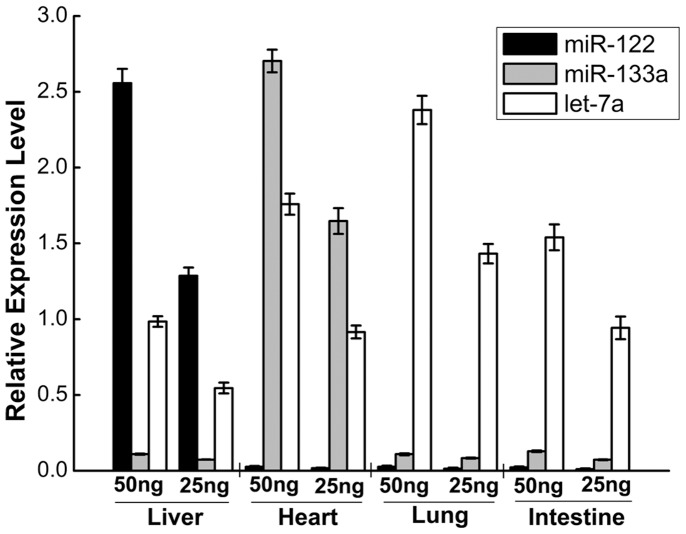
miRNA Expression Profile of Four miRNAs in Mouse Tissues. The miRNA expression values were normalized to the snRNA U6 expression data and are calculated with 2^−ΔΔ*C*q^ relative quantification. miR-122 and miR-133a are highly tissue specifically expressed in liver and heart, respectively. let-7a acted as a housekeeping microRNA. Each column represents the mean (± SD) of three measurements.

Optimization of the proposed miRNA quantification technique was required for practical applications. As well as experimental validation of the assay, it needed to be validated with biological samples. A miRNA expression map was created with the new assay by detecting the expression of 3 miRNAs in four BARBL/c mouse tissue samples (n = 5). After measuring the expression of the small nuclear RNA (snRNA) U6 as a housekeeping gene, the miRNA data were normalized by calculating the relative 2^−ΔΔ*C*q^ value. The expression patterns of the 3 miRNAs were in agreement with the previous observations. miR-122 and miR-133a were highly tissue specific and were expressed in the liver and the heart, respectively. miR-122 accounted for the domain of all mouse miRNAs found in the liver; miR-122 was almost undetected in all other tissues analyzed. miR-133a was expressed predominantly in the heart, and expressed at a low level in the liver. Additionally, let-7a acted as a housekeeping microRNA, and it was detected in all four mouse tissues (Figure 6).

## Discussion

The miRNAs play a crucial role in several biological processes and act as regulators of development, differentiation [19] and cell survival [6]–[8]. miRNAs function by pairing with mRNAs of protein-coding genes and regulating their post-transcriptional expression [3]. A great number of studies have indicated that miRNA expression profiles classify human cancers [20], [21]. Microarray is the most widely used high-throughput technique for the identification of a cancer-specific miRNA expression profile, but the low level of sensitivity is a disadvantage of this technique as it is difficult to amplify miRNA targets and can lead to false positive signals from closely related miRNAs and genomic sequences [22].

RT-qPCR is a powerful technique for quantifying gene expression in the life sciences and medicine as it is highly sensitive, accurate and simple [23]. And it is the most adaptive technique for the quantification of miRNAs used by the general research community. Several RT-qPCR assays have been established for miRNA quantification, and are currently made available by companies. Considering the similar small size of miRNAs with ordinary PCR primers, we proposed an optimal and convenient alternative process based on readily available techniques and materials. This assay allowed reverse transcription of the entire miRNA population from the same sample with the identical level of efficiency, followed by the amplification and quantification of cDNA with the simple, high-throughput SYBR® Green real-time PCR without using fluorochromic hybridization probes or LNA-modified oligonucleotides. The expression of a considerable number of human miRNAs have been detected using this technique in a variety of samples, including epidermal stem cells, bone marrow of leukemia patients, clinical cervical cancer samples and several cancer cell lines (unpublished data). Whether common samples which are easy to process (e.g. cell lines) or samples that are limited to obtain (e.g. clinical tissue samples), the new procedure had a dynamic monitoring range as well as exquisite sensitivity that have been proved by comparison with a purchasable kit. Compared to the TaqMan® small RNA assays (ABI), this new approach possessed an enhanced dynamic range of eight orders and the general detection limit of about 0.2 fM miRNA. These data establish that the new real-time PCR approach is particularly useful for the quantification of low copy number or under-expressed miRNAs because of the optimal efficiency of the 1–100 pg of total RNA templates.

Moreover, we used stem-loop RT primer instead of linear adaptor to make sure that this protocol is insensitive to double-stranded nucleic acid molecules. The spatial constraint of the stem-loop structure might prevent the RT primer from binding double-stranded genomic DNA molecules and enhance the thermal stability of the RNA-DNA heteroduplex [14]. The data clearly demonstrated that double-stranded genomic DNA did not affect the quantification of miRNAs. And we also observed a good concordance of the *C*_q_ values between the purified total RNA and heat-treated samples. This renders that the proposed new approach can analyze heat-lysed cells directly, eliminating the need for sample preparation. The method is also suitable for tiny samples of RNA that are difficult to isolate, a further advantage over existing assay techniques.

The possibility of the lower amplification efficiency with LNA-spiked primers would be supported by differences between the solution structure of a DNA:LNA helix and the structure of double-stranded DNA [17], [24]. The aforementioned findings prompted us to investigate amplification efficiency of the new assay. Comparison result as shown in Table 1 demonstrated higher-level efficiency of the present approach than the Exiqon miRCURY method. This finding suggests that the new assay could exert comparable specificity to LNA-spiked primers, but would not have a negative impact on amplification efficiency.

In this new assay, we used poly(U) instead of the traditional poly(A) as it can prevent the oligo(A) RT primer from binding the poly(A) tail of the mRNA. The RT reaction can achieve reverse transcription of miRNAs, mRNA and the internal control (U6) from the same sample in the same system, which has the advantage of keeping the identical reaction efficiency. It has been shown that polyuridylation of pre-miRNA might be inefficient due to the presence of the stem-loop structure [25], [26]. Thus, the negligible level and low polyuridylation efficiency of pre-miRNAs are unlikely to affect the quantification of the miRNA. Stem-loop/poly(A) RT primers (SL-poly(A)) can potentially be used for multiplex RT reactions of mRNA and miRNAs in the same run.

### Conclusions

The spatiotemporal expression patterns of miRNAs are important for the verification of their predicted function. There is an urgent need for a highly specific and simple method for quantification of miRNA. The proposed approach offers an alternative method for scientists to quantify multiple miRNA expression of the same sample. We are currently improving the approach, which is expected to increase the utility of this method.

## Materials and Methods

### Primers and Synthetic MicroRNA Molecules

The sequences of the 11 microRNA molecules selected for this assay were obtained from the miRBase Sequence Database Release 15 (www.mirbase.org). Synthetic miRNA molecules used for the validation of the method were purchased from Genepharma (Shanghai, China). Gene-specific primers were designed according to the miRBase Sequence Database and synthesized by Invitrogen (Beijing Office). Sequences of the mature miRNA molecules and the primers used in this study are shown in Table 2.

**Table 2:** Oligonucleotides used in this study.

Name	Sequence (5′ to 3′)
hsa-miR-455	GCAGUCCAUGGGCAUAUACAC
hsa-miR-126	UCGUACCGUGAGUAAUAAUGCG
hsa-miR-32	UAUUGCACAUUACUAAGUUGCA
hsa-miR-181a	AACAUUCAACGCUGUCGGUGAGU
hsa-miR-181b	AACAUUCAUUGCUGUCGGUGGGU
hsa-let-7a	UGAGGUAGUAGGUUGUAUAGUU
hsa-let-7b	UGAGGUAGUAGGUUGUGUGGUU
hsa-let-7c	UGAGGUAGUAGGUUGUAUGGUU
hsa-let-7d	AGAGGUAGUAGGUUGCAUAGUU
hsa-let-7e	UGAGGUAGGAGGUUGUAUAGUU
mmu-miR-122	UGGAGUGUGACAAUGGUGUUUG
mmu-miR-133a	GCUGGUAAAAUGGAACCAAAU
mmu-let-7a	UGAGGUAGUAGGUUGUAUAGUU
cel-miR-2	UAUCACAGCCAGCUUUGAUGUGC
real-time PCR primers	
miR-455-fw	GAACT**GCAGTCCATGGGCATA**
miR-126-fw	AGACC**TCGTACCGTGAGTAATA**
miR-32-fw	GCAAG**TATTGCACATTACTAAG**
miR-181a-fw	ACTGA**AACATTCAACGCTGTCGG**
miR-181b-fw	TGACG**AACATTCATTGCTGTCGG**
let-7a-fw^1^	CGTC**TGAGGTAGTAGGTTGTATA**
let-7b-fw	GTCG**TGAGGTAGTAGGTTGTGTG**
let-7c-fw	GTCG**TGAGGTAGTAGGTTGTATGGT**
let-7d-fw	TGAC**TAGAGGTAGTAGGTTGCATA**
let-7e-fw	ATGTC**TGAGGTAGGAGGTTGTATA**
mmu-miR122-fw	TGTCA**TGGAGTGTGACAATGGTG**
mmu-miR-133a-fw	ATTCA**GCTGGTAAAATGGAACC**
miR-2-fw	GCTAG**TATCACAGCCAGCTTTGA**
U6-Fw	CTCGCTTCGGCAGCACA
miRNA-rev	GCAGGGTCCGAGGTATTC
Reverser transcription primer	
Linear RT	GCGAGCACAGAATTAATACGACTCACTATAGGACGGCTTTTTTTTTTTTTTTVN
SL-poly(A)	*GTCGTATCCAGT* ***GCAGGGTCCGAGGTATTC*** *GCACTGGATACGAC*AAAAAAAAAAAAAAAAAAVN^2^

Stem-loop sequence is indicated in italic. Binding sequences for universal reverse primer are indicated in italic and bold. miRNA specific sequences of the forward primer are in bold.

1 hsa-let-7a and mmu-let-7a are highly conservative. They share the same forward primer.

2 V: A, C and G; N: A, C, G and T.

### Total RNA Isolation from Tissues and Cells

The human cervical carcinoma cell line SiHa was purchased from the American Type Culture Collection (ATCC, no. CCL-2) and cultured in a humidified atmosphere of 95% air, 5% CO_2_ using the recommended medium supplemented with 10% (v/v) fetal bovine serum (FBS).

Studies using human tissues were approved by the Institutional Ethical Committee in Chinese PLA General Hospital. The individual in this manuscript has given written informed consent (as outlined in the PLoS consent form) for the use of tissue samples and to publish these case details. All experiments involving animals were undertaken in accordance with the National Institute of Health Guide for the Care and Use of Laboratory Animals, with the approval of the Scientific Investigation Board of Chinese PLA General Hospital. Total RNA was extracted from the SiHa cell line, bone marrow of leukemia patients, and tissues of mice using TRIzol® reagent (Invitrogen, catalogue no. 15596026) according to the manufacturer’s instructions.

In order to assess the influence of genomic DNA, we used heat-lysed cells as a template for the RT reaction. The cell pellets were re-suspended in 100 µL 1× PBS, heated at 95°C for 5 min, and immediately chilled on ice before being added directly into the RT reaction.

Yeast tRNA was employed as an RNA carrier to provide a complex RNA background in RT reactions. It was purchased from Invitrogen (catalogue no.15401011).

The integrity and purity of the RNA was measured based on electrophoresis traces and A_260_/A_280_ value, respectively. RNA extraction was performed by two different operators simultaneously.

### Polyuridylation

Following the manufacturer’s instructions (New England Biolabs, catalogue no. M0337S), 10 ng of total RNA, certain amounts of the corresponding synthetic miRNA with 50 ng yeast tRNA, or heat-lysed cells was polyuridylated with UTP by poly(U) polymerase at 37°C for 1 h in a 20 µL reaction volume. After extraction with phenol/chloroform and precipitation in ethanol, the treated RNA was dissolved in diethylpyrocarbonate (DEPC)-treated water.

### Reverse Transcription

Reverse transcription was performed using the M-MLV RT kit (Invitrogen catalogue no. 28025013) according to the manufacturer’s instructions. The RT reaction was performed using treated total RNA and the RT primer SL-poly(A). The 12 µL RT reaction mixture contained 10 ng of treated RNA (or certain amounts of the corresponding treated synthetic miRNA), 0.5 µL of RT primer SL-poly(A) (5 µM) and 0.5 µL of 10 mM dNTP Mix (10 mM each). The mixture was heated at 65°C for 5 min and quick-chilled on ice. The contents of the tube were collected by centrifugation and 2 µL of DTT (0.1 M), 4 µL of 5× first-strand buffer, 1 µL of RNase inhibitor (40 U/µL, Qiagen) were added. The mixture was incubated at 37°C for 2 min, followed by the addition of 1 µL of M-MLV (200 U) and the incubation was continued for 50 min at 37°C. The reaction was inactivated by heating at 70°C for 15 min. The RT reaction was performed in triplicate to remove the RT outliers.

### Quantitative Real-time PCR

Real-time PCR was performed using the standard SYBR® Green PCR protocol (SYBR® Green Real-time PCR Master Mix, Toyobo, catalogue no. QPK-201) on a Rotor-Gene RG-3000A thermal cycler (Corbett Research), and each sample was analyzed in triplicate. The 20 µL PCR volume included 3 µL of RT product, 10 µL of 2× SYBR® Green real-time PCR Master Mix, and 1 µL of primer (forward and reverse, 5 µM each). The reactions were incubated at 95°C for 5 min, followed by 45 cycles of 95°C for 15 s, 55°C for 15 s, and 72°C for 20 s. The level of miRNA expression was measured using the *C*_q_ (quantification cycle) value. *C*_q_ is the fractional cycle number at which the fluorescence of each sample passes a fixed threshold. A synthetic miRNA molecule was used for calculation of the standard curve.

The 2^−ΔΔ*C*q^ method for relative quantification of gene expression was used to determine the level of miRNA expression. Δ*C*_q_ was calculated by subtracting the *C*_q_ value of U6 RNA from the *C*_q_ value of the miRNA of interest. The fold change was generated using the equation 2^−ΔΔ*C*q^.

## Supporting Information

Figure S1 **Sensitivity of propsed assay compared with the TaqMan assay.** (A) Amplification plot of synthetic miRNAs hsa-miR-455, 181a, 181b and 126. Target input ranged over eight orders of magnitude (0.3–0.5 fM to 3–5 nM). (B) Stardard curve of the four miRNAs of the new proposed assay and TaqMan method. Curves of the new assay were straight lines (*R^2^* = 0.9932–0.9938) with slope of −3.378 to −3.391 (PCR efficiency = 97.2–97.7%) over eight orders of magnitude of the template. Curves of TaqMan method were also straight lines (*R^2^* = 0.9919–0.9925) with slope of −3.432 to −3.482 (PCR efficiency = 93.7–95.6%) over seven orders of magnitude of the template. (C) The TaqMan method showed sensitivity limit of 3–5 fM multiple synthetic miRNAs, while the sensitivity limit of the new assay turned out to be 0.3–0.5 fM multiple synthetic miRNAs. Each column represents the mean (± SD) of three measurements.
(TIF)
