# Supplementary figures and images for: A Facile and Specific Assay for Quantifying MicroRNA by an Optimized RT-qPCR Approach
Source: PLoS One. 2012 Oct 5;7(10):e46890. doi: 10.1371/journal.pone.0046890 (PMC3465266; doi:10.1371/journal.pone.0046890)

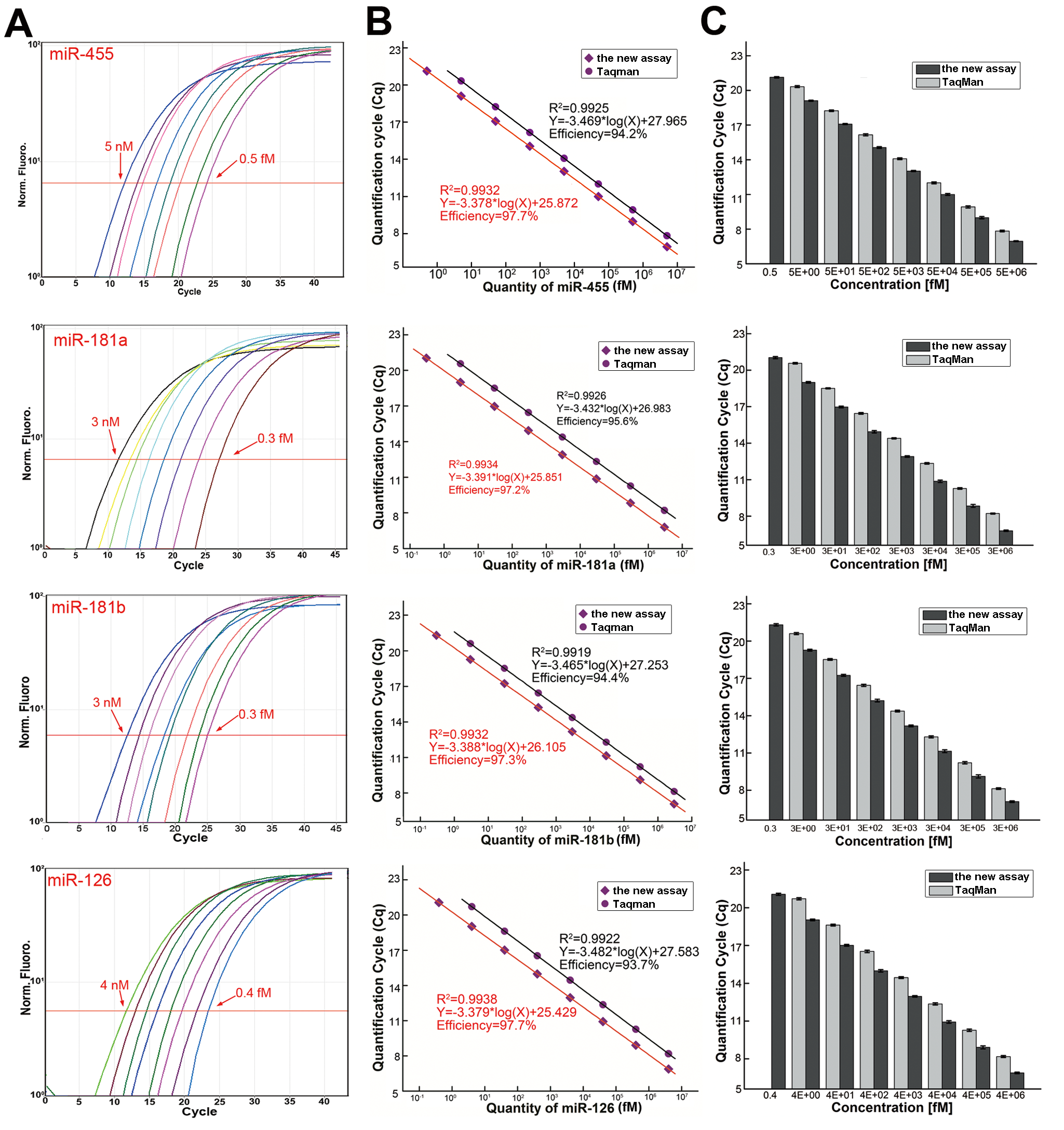

Supplement: Figure S1 — Sensitivity of propsed assay compared with the TaqMan assay. (A) Amplification plot of synthetic miRNAs hsa-miR-455, 181a, 181b and 126. Target input ranged over eight orders of magnitude (0.3–0.5 fM to 3–5 nM). (B) Stardard curve of the four miRNAs of the new proposed assay and TaqMan method. Curves of the new assay were straight lines (R2 = 0.9932–0.9938) with slope of −3.378 to −3.391 (PCR efficiency = 97.2–97.7%) over eight orders of magnitude of the template. Curves of TaqMan method were also straight lines (R2 = 0.9919–0.9925) with slope of −3.432 to −3.482 (PCR efficiency = 93.7–95.6%) over seven orders of magnitude of the template. (C) The TaqMan method showed sensitivity limit of 3–5 fM multiple synthetic miRNAs, while the sensitivity limit of the new assay turned out to be 0.3–0.5 fM multiple synthetic miRNAs. Each column represents the mean (± SD) of three measurements. (TIF) [file pone.0046890.s001.tif]
